# Survivable Deployments of Optical Sensor Networks against Multiple Failures and Disasters: A Survey

**DOI:** 10.3390/s19214790

**Published:** 2019-11-04

**Authors:** Yongjun Zhang, Jingjie Xin

**Affiliations:** State Key Laboratory of Information Photonics and Optical Communications, Beijing University of Posts and Telecommunications, Beijing 100876, China; jingjiex@bupt.edu.cn

**Keywords:** network survivability, disaster-resilience, optical sensor networks (OSNs), optical sensing, optical networks, *k*-node (edge) sensing connectivity, artificial intelligence (AI)

## Abstract

Optical sensing that integrates communication and sensing functions is playing a more and more important role in both military and civil applications. Incorporating optical sensing and optical communication, optical sensor networks (OSNs) that undertake the task of high-speed and large-capacity applications and sensing data transmissions have become an important communication infrastructure. However, multiple failures and disasters in OSNs can cause serious sensing provisioning problems. To ensure uninterrupted sensing data transmission, survivability has always been an important research emphasis. This paper focuses on the survivable deployment of OSNs against multiple failures and disasters. We first review and evaluate the existing survivability technologies developed for or applied to OSNs, such as fiber bus protection, self-healing architecture, and 1 + 1 protection. We then elaborate on the disaster-resilient survivability requirement of OSNs. Moreover, we propose a new *k*-node (edge) sensing connectivity concept, which ensures the connectivity between sensing data and users. Based on *k*-node (edge) sensing connectivity, the disaster-resilient survivability technologies are developed. The key technologies necessary to implement *k*-node (edge) sensing connectivity are also elaborated. Recently, artificial intelligence (AI) has developed rapidly. It can be used to improve the survivability of OSNs. This paper details potential development directions of survivability technologies of optical sensing in OSNs employing AI.

## 1. Introduction

In recent years, new industries such as cloud computing, big data, data center, virtual/augmented reality (VR/AR), 5G, artificial intelligence (AI), Internet of Things (IoT), and optical fiber sensing have emerged. These developments have changed our way of life and simplified the completion of tasks that were difficult in the past. For example, the AI group in Tencent Corporation uses deep learning to successfully locate abducted children simply using photos from their childhoods [1]. In the past it was difficult to realize cross-age face recognition. Deep learning has benefitted society and humanity. Well-known companies such as Google, YouTube, Facebook, Alibaba, and Tencent have built a notable number of large-scale data centers to support those emerging industries [2,3]. Data centers are designed to host massive storage and computing resources, and to support computing-intensive and storage-intensive applications. Requirements for data centers to deliver applications and services at high-speed and high-throughput have become greater. Therefore, it is necessary to achieve high-speed and large-capacity communication among data centers located in different geographical locations. Optical interconnection, which provides flexible interconnection reconfigurations for various topologies and supports transparent, large-capacity, and high-speed data transmission, is widely used [4,5,6,7,8]. Data center networking has evolved from hybrid optoelectronic networking to flex-grid optical networking [9,10,11,12]. In addition, optical fiber sensing that integrates communication and sensing functions plays a more and more important role [13,14,15,16]. It provides sensing solutions with optical performance for almost all kinds of applications and environments, such as monitoring of oil fields and large civil engineering structures, as well as natural environments [17,18,19]. In [20], the authors gave an overview of optical sensing technology for electromagnetic field measurement. They analyzed the principles of several types of sensors, including the probe-based Faraday effect, magnetostrictive materials, and magnetic fluids, discussed each advantage and disadvantage, and reviewed future outlooks on the performance improvement of sensors. In [21], the authors gave an overview of recent advances in optical fiber acoustic sensing systems in the domains of military defense, structural health monitoring, and petroleum exploration and development. It can be seen that optical fiber sensing is widely used. 

To improve quality of service (QoS) and guarantee uninterrupted traffic transmission, survivability remains an important part of network design. It represents the ability of a network to fulfill its mission of data transmission, in a timely manner, when threatened by attacks or large-scale natural disasters. In the case of an unavoidable link cut or a network node becoming ineffective due to misconfiguration or natural disaster, network survivability needs to quickly and effectively resume the interrupted traffic, thus keeping damage to a minimum. Due to wide distribution and high severity, natural disasters are big threats to the normal operation of optical sensor networks (OSNs). Therefore, the survivability of optical sensing and optical communication in OSNs in times of disaster is attracting wide-spread attention [22,23,24,25,26]. Moreover, optical sensing can help improve the survivability of optical networks [27,28]. In [27], the authors proposed optical chaos and hybrid wavelength division multiplexing/time division multiplexing (WDM/TDM) based on large capacity quasi-distributed sensing networks. With WDM/TDM technology, hundreds of sensing units could be multiplexed in multiple sensing fiber lines. This sensing network could achieve real-time fiber fault monitoring. In [28], the authors used a distributed optical fiber sensor to improve the optical fiber cable condition monitoring system. A series of survivability technologies designed to ensure optical sensing and optical communication resist link/node failures already exist. For example, the classic p-cycle scheme ensures that there is at least one available light path between any node pair located in this cycle after a random single failure [29]. The traditional survivability techniques can be divided into two categories: protection schemes and restoration schemes. The protection scheme reserves backup resources for working flows and only needs to conduct protection switching at the source and destination nodes when failures occur [30]. The restoration schemes, such as link-based restoration and path-based restoration, attempt to establish recovery channels for interrupted working flows using the remaining available network resources after failures [31]. 

Both the protection and restoration scheme rely on network connectivity. In mathematics and computer science, network connectivity is one of the basic concepts of graph theory, which asks for the minimum number of nodes or edges that need to be removed to disconnect the remaining nodes from each other [32]. Since network connectivity, indicated by vertex (edge) connectivity, has a fixed upper bound for a given topology, the connectivity between a node pair can be easily destroyed by disasters. Merely relying on network connectivity to realize uninterrupted traffic transmission will cause bottlenecks. Fortunately, for some emerging services, such as high-definition TV, web searching, scientific computing, and cloud service, the required data can be replicated and maintained in multiple data centers through synchronization technology [33,34,35]. Therefore, service providing is no longer confined to one particular data center and any data center that hosts the required service can be designated a service provider. Moreover, the service can dynamically migrate to multiple data centers according to users’ demands. For optical data center networks, traditional end-to-end connections are gradually replaced by end-to-content connections. The end-to-content connection means that the destination node is not fixed and can be any reachable data center where the required service is hosted. The user can obtain the required service along any available end-to-content connection. Even if a natural disaster breaks the optical data center networks into several disconnected parts, the service will not be interrupted as long as one reachable data center in each part remains. This new kind of connectivity is called content connectivity, which is defined as the reachability of the content from any point of a data center network [36]. It no longer merely ensures connectivity between source–destination node pairs but guarantees connectivity between users and their required services. Moreover, the *k*-node (edge) content connectivity concept, which indicates the minimum number of elements (nodes or edges) that need to be removed to disconnect the remaining nodes from the required service, was proposed [37]. 

In contrast to content connectivity and *k*-node (edge) content connectivity, a new *k*-node (edge) sensing connectivity concept, which ensures the connectivity between sensing data and users, was proposed. Based on *k*-node (edge) sensing connectivity, disaster-resilient survivability technologies have been developed. Recently, AI has become a hot topic and research focus. It has been applied in many aspects of optical networks, including failure localization and anomaly detection, routing and resource allocation, modulation level recognition, optical interconnection, network control and management, and quality of transmission (QoT) estimation [38,39]. This paper first reviews and evaluates the existing survivability technologies that have been developed for or can be applied to OSNs, such as fiber bus protection, self-healing architecture, 1 + 1 protection and extension, p-cycle, the photonic millimeter-wave bridge scheme, p-polyhedron, multi-path protection, and restoration. Then, the *k*-node (edge) sensing connectivity concept is elaborated. Based on *k*-node (edge) sensing connectivity, disaster-resilient survivability technologies were developed. Moreover, the key technologies for implementing *k*-node (edge) sensing connectivity in elastic optical networks are elaborated. Finally, this paper explores the potential development direction of survivability techniques of optical sensing and optical communication in OSNs employing AI against multiple failures and disasters. 

## 2. Existing Survivability Technologies in OSNs

To improve the QoS of optical sensing and optical communication against node/link failures, many survivability techniques have been developed. The traditional survivability techniques can be divided into two categories: protection schemes and restoration schemes. The protection scheme needs to conduct resource reservation before failures. Since it avoids the complex routing and signaling processes, the protection scheme has a short recovery time. Moreover, the protection scheme can also be classified as dedicated and shared protection. Dedicated protection only assigns backup resources to specified working traffic, but shared protection allows multiple backup paths sharing the spectrum resource on common links as long as their working paths do not fail simultaneously. Compared with dedicated protection, shared protection has higher spectrum efficiency. The restoration scheme attempts to establish a new recovery path for the interrupted service by employing the remaining available resources after a failure. It needs to conduct routing and signaling processes to establish a new light path. Therefore, the restoration scheme requires more time than the protection scheme. Moreover, the restoration scheme may fail when the remaining available resources are inadequate. In this section, we first elaborate the traditional survivability techniques designed for optical sensing, such as the fiber bus protection scheme [40,41,42,43,44,45] and self-healing architecture [46,47,48,49,50,51,52,53]. Then, we elaborate the traditional survivability techniques that can be applied in OSNs, such as 1 + 1 protection and extensions [54,55,56,57,58,59,60,61,62,63,64,65,66,67,68,69,70,71,72], the photonic millimeter-wave bridge scheme [73,74,75,76,77,78], the p-cycle scheme [79,80,81,82,83,84,85,86,87,88,89,90,91,92,93,94,95,96,97], the p-polyhedron scheme [98,99,100,101,102,103,104,105,106,107,108,109,110], the multipath protection scheme [111,112,113,114,115,116,117,118,119,120], and the restoration scheme [121,122,123,124,125,126]. Finally, we conduct performance analysis for the above-mentioned survivability technologies.

### 2.1. The Fiber Bus Protection Scheme

**Figure 1:**
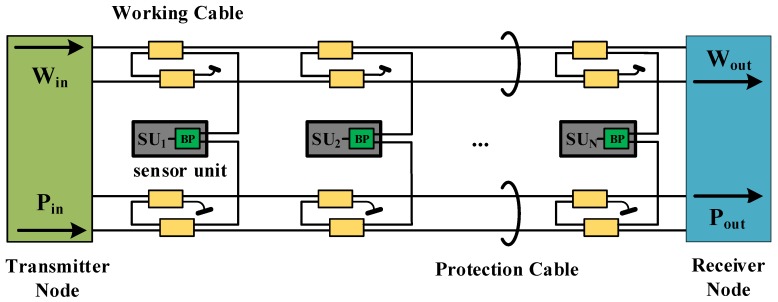
The fiber bus protection scheme [40]; BP: branching point, SU: sensor units, W_in_: working cable input, W_out_: working cable output, P_in_: protection cable input, and P_out_ protection cable output.

The optical sensor network is used to transfer sensing data through fiber links. It is easily damaged with failures and disasters. Multiple survivability techniques designed for optical sensor networks already exist. The fiber bus protection scheme, which can withstand failures at one or more points, is a classic. As presented in Figure 1, the fiber bus protection scheme uses two fiber cables to achieve sensing path protection. When the branching point (BP) is implemented by a passive l × 2, this scheme belongs to dedicated protection. When the BP is implemented by a l × 2 photonic switch, this scheme belongs to shared protection. The fiber bus protection scheme has been widely researched. In [40], the authors presented the optical fiber bus network for the multiplexing of sensors. The network can survive at least one failure at any location. In [41], a symmetrical dual-fiber bus that uses dedicated-liner or dedicated-path protection switching in response to a failure was developed for optical sensing. In [42], the authors proposed a mathematical model for a WDM self-healing optical fiber bus network to interconnect an array of sensors. The network can determine its own sites of failure from network management information without requiring external resources. In [43], the authors proposed a multi-bus fiber-optic sensor network, which could multiplex different types of fiber sensors, locate the failure position, and recover the sensing services of sensors in a network immediately and automatically to keep them working without being interrupted. In [44], the authors reported a self-healing fiber bus network for the wavelength multiplexing of sensors. The network can recover service after one or more failures by dedicated line or dedicated path protection. In [45], the authors reported a resilient WDM fiber bus network with interconnected sensors. The network self-diagnosed and identified the failed constituent(s) from the patterns of surviving end-to-end connections at its operating wavelengths. 

### 2.2. Self-Healing Architecture

**Figure 2:**
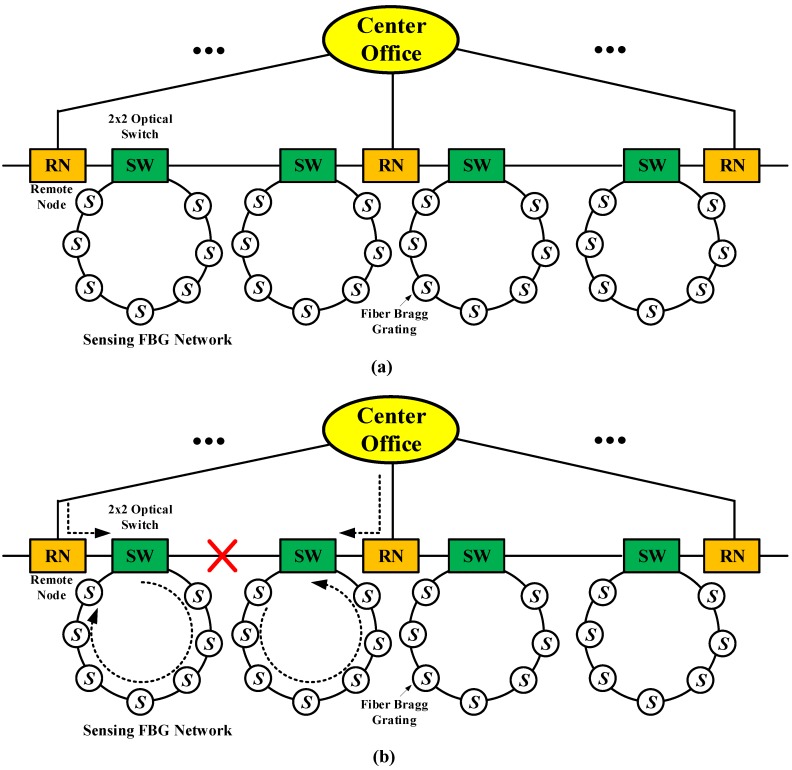
Self-healing architecture: (**a**) FBG sensor network; (**b**) failure in the bus subnet [46] RN: remote nodes; *S:* fiber Bragg grating, and SW: 2 × 2 optical switch.

Self-healing architecture is another important survivability technique for the optical sensor network. As presented in Figure 2, self-healing architecture consists of the fiber Bragg grating (FBG) network and a central office. This self-healing architecture offers a survival function during link failure by reconfiguring the fiber network with remote nodes (RNs) that are responsible for performing self-healing functions if link failure occurs in the lower level network. Self-healing architecture has also been widely researched. In [46], the authors presented a fiber-laser-based sensor network with a self-healing function. The survivability of fiber-laser-based sensor networks was enhanced by adding switches in the self-healing ring architecture. In [47], the authors proposed self-healing architecture for FBG sensor networks. They designed remote nodes using simple optical switches to check the breakpoint and reconfigure the FBG network if any links failed. In [48], the authors proposed two large-scale self-healing architectures for FBG sensor networks. They designed branch nodes using simple optical switches and couplers to reconfigure the FBG sensing subnets if any links failed. In [49], the authors proposed and demonstrated a novel 3-D ring-mesh sensing system with a comprehensive self-healing function. The proposed ring-mesh topology is constructed by 2 × 2 optical switches to link the ring-based subnets in a meshed architecture. In [50], the authors proposed and demonstrated novel FBG-based passive sensor architecture that could be used to protect the fiber cut and monitor multiple sensors simultaneously. In [51], the authors proposed an FBG sensor system with two-level ring architecture. The survivability of an FBG for a multipoint sensor system is enhanced by adding remote nodes and optical switches to the two-level ring architecture. In [52], the authors presented a novel star-ring-bus sensor system. The main trunk of the proposed sensor system is a star topology and the sensing branches comprise a series of bus subnets. Any weakness in the reliability of the sensor system is overcome by adding remote nodes and switches to the ring and bus subnets. In [53], the authors presented hybrid star-ring architecture for a highly reliable FBG sensor system. The main trunk of the proposed sensor network is a star topology, and the sensing branches comprise a series of concatenated ring subnets. 

### 2.3. Pre-Configured Protection Schemes

#### 2.3.1. The 1 + 1 Protection Scheme and Extensions

**Figure 3:**
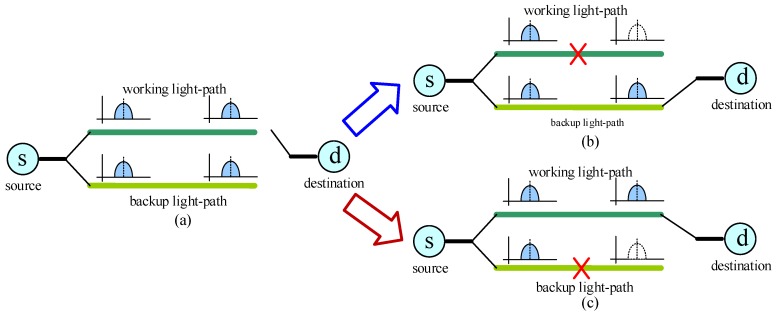
The 1 + 1 protection scheme: (**a**) 1 + 1 protection configuration; (**b**) the failure occurs at the working light path; (**c**) the failure occurs at the backup light path; s: source node and d: destination node.

The 1 + 1 protection scheme is the most classic and simplest protection scheme for optical networks. It can also be applied into OSNs. As presented in Figure 3, the 1 + 1 scheme uses two shared risk link group (SRLG) disjoint light paths to transfer data simultaneously. If a failure occurs at an intermediate node or link of a light path, the source and destination nodes of this light path only need to conduct protection switching. SRLG is an important concept for survivability. It represents a group in which all links will fail simultaneously as a result of a common failure [54,55]. For example, all links in a seismic zone can make up an SRLG because all these links will be destroyed when an earthquake occurs in this seismic zone. The 1 + 1 protection scheme has been studied by a large number of researchers involving resource efficiency, recovery time, flexibility, etc. In [56], the authors developed a light path provisioning algorithm for wavelength-continuous optical transport networks under 1 + 1/1:1 service protection. They showed that a limited number of add/drop ports and limited transmitter tunability were sufficient to ensure that a network fully utilizes its wavelength capacity. In [57], a transmitter was modified to provide two signals without excess insertion loss. Using this transmitter, optical protection switching was demonstrated under 10 Gb/s non-return-to-zero transmission and a protection time of 5 ms was obtained. In [58], an innovative transmission technique enabling signal overlap was introduced for spectrally efficient 1 + 1 protection. The results showed that the proposed technique reduced the overall amount of occupied spectrum resources. In [59], the authors used a practical all-optical XOR network coding to leverage the 1 + 1 protection in transparent WDM optical networks. This network coding-assisted protection solution paved the way for reducing the backup capacity. The 1 + 1 scheme is also applied in optical fiber access networks. In [60], the authors compared the reactivation times of different 1 + 1 protection scenarios, and then they proposed hardware modifications to reduce protection time.

Although the 1 + 1 protection scheme is the most basic and simplest protection scheme, it consumes too many spectrum resources. To improve the resource efficiency of the 1 + 1 protection scheme, the 1:1, 1:N, and M:N protection schemes were developed. Compared with the 1 + 1 protection scheme, the 1:1, 1:N, M:N protection schemes allow the backup resources to be shared by multiple working flows. In [61], the authors extended single failure to dual failure recovery for an elastic optical network (EON) with 1 + 1:1 network protection. In [62], the authors demonstrated an reconfigurable optical 1:N protection system using the branch state of LiNbO/sub 3/optical switches. In [63], the authors presented a 1:N protection scheme based on the cyclic property of an array waveguide grating and a specific connection pattern among the optical network units (ONUs). The proposed scheme required more fiber links among the ONUs than previous schemes, but the network resource demands were greatly reduced and the protection performance was improved. In [64], the authors presented a theoretical study on the dynamic performance requirements for the successful application of a proposed 1 for N wavelength protection scheme to wavelength routed trunk and branch type optical networks. In [65], the integrated MEMS 1:N protection switch, scalable to M:N protection, was proposed, which allows monitoring of the redundant network element during normal operation and of the failed network element during protection for diagnostic purposes. In [66], the authors provided the mathematical formulation to evaluate the availability/unavailability of protected optical connections for the general M:N protection scheme in WDM optical networks. In [67], the authors used shared protection for multicast demands where each light-tree was protected from any single link failure in both directions by having a backup path that is link-disjointed to the path from the source to each destination on the primary tree. The failure probability of each link is already considered in the 1 + 1, 1:1, 1:N, and M:N protection schemes. In [68], the authors proposed shared-path protection with joint failure probability constraint in flexible bandwidth optical networks. They developed three different algorithms and showed that the minimum free spectrum block (MFSB) consumption algorithm not only achieved better performance in terms of blocking probability, spectrum consumption, spectrum redundancy, and hop counts, but also guaranteed a joint failure probability lower than the maximum shared spectrum block consumption algorithm. In [69], the authors proposed spectrum-aware survivability strategies with failure probability constraints under static traffic in flexible bandwidth optical networks. An integer linear program (ILP) model and two algorithms were developed. In [70], the authors proposed a sub-tree based optical multicasting scheme in elastic optical networks. In [71], they proposed the link importance incorporated failure probability measuring solution for multicast light-trees. In [72], the authors proposed distributed sub-light-tree-based multicast provisioning with a shared protection scheme. Each source–destination pair of a primary DSLT was protected by a link-disjoint backup path against any link failure. 

#### 2.3.2. The Photonic Millimeter-Wave Bridge Scheme

**Figure 4:**
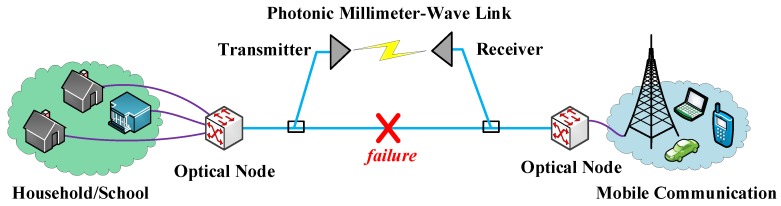
The photonic millimeter-wave bridge scheme.

Being similar to the 1 + 1 protection scheme, the photonic millimeter-wave bridge scheme uses millimeter-wave technology to recover the interrupted traffic, especially in passive optical networks (PONs). Since the topological type of PON is a star structure, it is impossible to recover the interrupted traffic merely by relying on the PON itself. A millimeter-wave wireless link transmits data at high speed from one point to another point. It serves as a bridge and represents the seamless and transparent integration of the wireless link into optical networks. In Figure 4, when a failure occurs at the fiber link, a millimeter-wave wireless link is established to recover the interrupted traffic. 

The photonic millimeter-wave bridge scheme has been widely studied. In [73], the authors proposed millimeter-wave bridge-based RF frequency doubling to overcome accidental fiber cuts in optical access networks with lower high-frequency devices less sensitive to fiber chromatic dispersion. In [74], record-speed wireless data bridging at 220 GHz was demonstrated with a 20 Gbit/s non-return-to-zero (OOK) or a 9 Gbit/s orthogonal frequency division multiplexing (OFDM) signal. In [75], the authors focused on a resilient optical and MMW wireless transmission system for a novel span protection scheme in disaster recovery; the key enabling technologies of optical and wireless sub-systems and devices were shown. In [76], the authors presented an end-to-end intermediate frequency-over-fiber-based fiber-wireless bridge system, which can be applied to applications that require high agility and mobility. In [77], the authors demonstrated novel digital radio-over-fiber architecture able to transport multiple compressed digitized RF services using both optical fiber and wireless millimeter-wave links and which shows 3-times higher efficiency than a common public radio interfaces. In [78], the author showed that transparent waveform transfer can make seamless integration of wireless and wired links, which can provide resilient and low-latency networks.

#### 2.3.3. The P-Cycle Scheme

**Figure 5:**
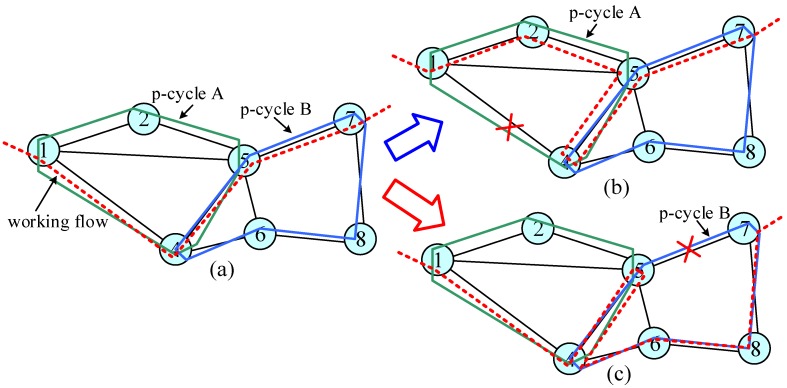
The p-cycle scheme: (**a**) p-cycles configuration; (**b**) a failure occurs at p-cycle A; (**c**) a failure occurs at p-cycle B.

Although 1:1, 1:N, and M:N protection schemes can improve resource efficiency, the resource efficiency of these schemes is still very high, especially when many working flows are accommodated. In 2000, W.D. Grover proposed the p-cycle structure with the features of ring-like speed and mesh-like capacity [29]. They showed that p-cycle has the highest protection efficiency and the lowest resource redundancy against single failure. In other words, p-cycle is the optimal protection structure against single failure in mesh networks. As presented in Figure 5, two p-cycles were designed to protect a working flow. When a failure occurs at any link along the working flow, the interrupted working flow can be restored by the corresponding p-cycle. At present, the research on the p-cycle scheme involves cycle selection, node protection, spectrum efficiency, dual-failure protection, etc.

**Cycle Selection**: For the p-cycle scheme, the first problem is how to search the required cycles. In [79], the authors proposed a heuristic approach to iteratively select and refine a set of p-cycles. The results showed that the proposed approach was within 3.5% redundancy difference of the optimal solution with very fast computation time even for large networks. In [80], the authors developed a new ILP model and a related heuristic method for failure-independent path-protecting (FIPP) p-cycle design that produced network designs with much faster runtimes. In [81], the authors proposed a new heuristic approach and a new evaluation metric to solve the p-cycle protection problem that simultaneously optimized cost, protection, length, and fairness of solutions in a mono-objective approach. In [82], the authors proposed a novel path-protecting p-cycle heuristic algorithm for survivable WDM network design. The p-cycle based path protection for span and node failure recovery was capacity efficient and robust. In [83], the authors proposed the attached node-based cycle generation algorithm for pre-computation of candidate cycles and three algorithms for light-path-protecting p-cycle selection for a protected working light-path envelope. 

**Node Protection**: Although the p-cycle scheme was designed for link failure, it can also be used to achieve node protection. In [84], the authors showed that the transiting path affected by the node failure is inherently restorable by ordinary p-cycle switching actions whether the respective two-hop segment was on-cycle, straddling, or partially on-cycle and partially straddling. In [85], the authors showed that high levels of node failure protection can be achieved with an ordinary set of p-cycles, which are designed in such a way that every demand that transits a node in a straddling manner is also intercepted at points upstream and downstream on its route by some other p-cycle. In [86], the authors developed capacity optimization models to support 100% restoration of transiting flows through failed nodes. Only a very small additional spare capacity was required to achieve both 100% span and intermediate node-failure restorability.

**Spectrum Efficiency**: Improving the spectrum efficiency of the p-cycle scheme is an important research point. In [87], the authors studied how the efficiency of a p-cycle network increased under joint optimization of the working path routes with p-cycle placement. In [88], the authors showed how grooming decisions can be directly integrated into an overall p-cycle network design for more efficiency. In [89], the authors introduced the concept of differential capacity p-cycles as an enhancement to span protecting p-cycles. In [90], the authors provided a true comparison of span-protecting p-cycles with FIPP p-cycles, from the capacity efficiency perspective. In [91], the authors developed an approach that was a combination of GA methods with ILP and used it to create and solve p-cycle network design problems involving 200 or more nodes. In [92], the authors proposed a new p-cycle-based dynamic multicast protection scheme that achieved both fast restoration and high capacity efficiency. In [93], the authors evaluated the performance of the flooding-based distributed cycle pre-configuration utilizing a proposed removal of loop back (RLB) approach with various classical p-cycle enumeration algorithms and allocation strategies. 

**Dual-failure Protection**: The p-cycle scheme can also be used to achieve dual-failure protection. In [94], the authors developed a new linear programming model for the design of p-cycle networks that have a specified minimum dual-failure restorability level. In [95], the authors proposed a new optimization solution method for the design of dual failure survivable p-cycle based WDM mesh networks that guarantee quantified service availability under different dual failure probability distributions. In [96], the authors investigated a novel p-cycle-based dedicated protection architecture that could be used to enable high levels of dual failure restorability for select services. In [97], the authors proposed methods for achieving high dual-failure restorability in p-cycle networks optimally designed only to withstand all single failures or have minimized amounts of additional capacity for dual-failure considerations.

#### 2.3.4. The Pre-Configured K-Regular and K-(Edge)-Connected (*K&K*) Structure Scheme

**Figure 6:**
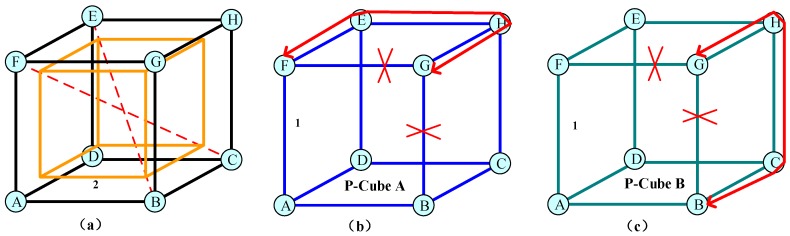
The p-cube scheme: (**a**) p-cube configuration; (**b**) traffic recovery in p-cube A; (**c**) traffic recovery in p-cube B [96].

Although the p-cycle scheme can be used to achieve multi-failure protection, it consumes too many spectrum resources. There is a pressing need to find a new protection structure that has higher spectrum efficiency and lower resource redundancy against multiple failures. In theory, p-cycle is the optimal protection structure against single failure. P-cycle is a two-regular and two-connected structure. Based on this, the pre-configured *K*&*K* structures to achieve multi-failure protection were proposed. It can be proved that the pre-configured *K*&*K* structure was the optimal protection structure against multiple failures. As presented in Figure 6, two p-cubes were designed for dual-link failures. When two failures occurred on any two links, the interrupted working flow could be restored by the two cubes. The pre-configured *K*&*K* was researched in WDM networks and elastic optical networks. In [98], the authors proposed *K*&*K* structures for providing protection against multiple failures in optical transport networks. In [99], the authors applied *K*&*K* structures to ultra-high capacity optical networks. They conducted theoretical analyses and showed that pre-configured *K*&*K* structures could reach the lower bound of logical redundancy. In [100], the authors presented an OpenFlow-based *K*&*K* structure configuration mechanism for elastic optical networks against concurrent multi-faults. In [101,102], a preconfigured *k*-edge-connected structure (p-kecs) was proposed in WDM networks. Many preconfigured protection structures have been proposed based on the pre-configured *K*&*K* structure, such as a pre-configured polyhedron (p-poly) structure, pre-configured multi-dimensional protection (p-MDP) structure, p-cube structure, preconfigured ball (p-ball) structure, and pre-configured prism structure.

**P-Poly Structure**: P-poly is a kind of pre-configured *K*&*K* structure. In [103], the authors proposed a novel protection scheme based on a p-poly structure against multi-link failures in high capacity and large-scale optical transport networks. In [104], the authors developed an efficient ILP construction model and two resource allocation schemes for p-poly. 

**P-MDP Structure**: The P-MDP structure was proposed to resist multi-link failures. In [105], the authors proposed a pre-configured multi-dimensional protection (p-MDP) structure against simultaneous m-link failures in high capacity and large-scale optical transport networks. In [106], a k-dimensional protection structure (KDPS) was proposed against *k* − 1 failures. Two greedy algorithms were proposed to construct KDPS in static and dynamic optical networks, respectively.

**P-Cube/P-Ball/P-Prism Structures**: The p-cube/p-ball/p-prism structures were proposed to resist dual-link failures. In [107], the authors proposed the p-cube structure in multi-dimensional node-based optical networks. Compared with p-cycle, p-cube fully used the node connectivity topology to employ fewer protection links against simultaneous dual-link failures. In [108], the authors proposed p-cube to deal with multi-link failures in a server-centric data center. The results showed that p-cube had better performance than p-cycle at the resource utilization rate and protection rate. In [109], the authors proposed a p-ball protection method for dual-link failures in optical mesh networks. The p-ball protection was better in terms of both construction cost metrics and operation cost metrics (such as average hop and protection resource utilization ratio). In [110], the authors proposed the p-prism structure against simultaneous dual-link failure and proved that it used less protection links than p-cycle.

#### 2.3.5. The Multi-Path Protection Scheme

The multi-path protection (MPP) scheme is another important survivability technology that can be used to resist multiple failures. For the MPP scheme, a data stream is split into multiple low-rate streams, each of which is routed and assigned to a separate path. At present, research about the MPP scheme mainly focuses on multipath configurations. Besides, other research focuses on improving the spectrum efficiency or extending the application range of the MPP scheme. 

**Survivable Multipath Configuration**: For the MPP scheme, the first important research is to conduct routing and spectrum allocation for each light path. In [111,112], the authors defined the static survivable multipath routing and spectrum allocation (SM-RSA) problem in OFDM-based flexible optical networks. In [113], the authors proposed a new survivable MPP scheme in OFDM-based optical networks and studied the static SM-RSA problem. In [114], the authors proposed a novel energy efficient multipath-based survivability scheme against single link failure in a static traffic scenario. In [115], the authors studied the protection schemes for MPP to ensure 100% restoration against single-link failures. In [116], the authors proposed an OpenFlow-based multipath protection scheme for elastic optical networks. 

**Optimization for Survivable Multipath**: To improve the spectrum efficiency of the MPP scheme, shared protection can be applied among multi-paths. In [117], the authors proposed a spectrum-efficient shared-protection MPP (S-MPP) scheme against multiple failures in a flexible-grid optical network. The MPP scheme can also be applied in virtual optical network (VON) mapping and network virtualization. In [54], the authors proposed a survivable routing and resource assignment scheme with MPP and SRLG in VON to get less resource consumption and enough protection against physical failure. In [118], the authors proposed a survivable multipath routing and spectrum assignment scheme for anycast traffic in CDNs against multiple node failures for less resource consumption and enough protection. In [119], the authors proposed a multipath protection-based virtual network function placement and scheduling scheme in elastic optical data center networks. It outperformed conventional protection methods in terms of spectrum and computing resource utilization as well as blocking probability. In [120], the authors proposed a survivable multipath virtual network embedding scheme against multiple failures. 

### 2.4. The Restoration Scheme

Although the restoration scheme tries to establish a new light path for the interrupted service employing the remaining available resources after failures, it cannot guarantee 100% recovery. Compared with the protection scheme, the restoration scheme does not need to reserve spectrum resources in advance so it usually requires more recovery time. For natural disasters, it is not a disaster-resilient survivability technology, especially when a large number of services need to be recovered after natural disasters. The inadequate available resources may lead to network paralysis. In [121], the authors proposed a fuzzy fault set (FFS)-based multi-link faults restoration algorithm. Experimental results showed that it had better performance compared with the extended limited perimeter vector matching protocol and could improve the restoration success rate under multi-link faults. In [122], the authors proposed a dynamic restoration scheme for EONs based on the SDN framework. The proposed scheme contemporarily exploits centralized path computation and node configuration to avoid contentions during the recovery procedure with the final aim of minimizing the recovery time. In [123], three network operator policies were proposed to yield different restoration mechanisms according to different inter-domain failure exchanges. In [124], the authors proposed a restoration-based survivability strategy, which combines the benefits of both cloud service relocation and service differentiation concepts. In [125], the authors exploited wavelength converters in GMPLS-based wavelength switched optical networks to reduce resource contentions during light-path restoration. In [126], the authors examined dynamic optical service restoration on a large-scale ROADM network. They showed that with dynamic restoration, full service restorability can remain achievable even in the face of four or more concurrent fiber cuts. 

### 2.5. The Performance Comparisons

Table 1 presents the performance comparisons among traditional survivability technologies in terms of survival capability, recovery time, resource efficiency, and complexity. The pre-configured *K*&*K* structure scheme and the MPP scheme were designed for multi-failure protection. Compared with the MPP scheme, the pre-configured *K*&*K* structure scheme had high spectrum efficiency. Since the self-healing architecture and the restoration scheme need to conduct signaling processes, more time will be consumed. Besides, the p-cycle scheme and the pre-configured *K*&*K* structure scheme both need to search the required protection structure, so that the implementation complexity is high. Since the multi-path protection scheme and the restoration scheme need to conduct routing and spectrum allocation, the implementation complexity is also high. For the 1 + 1 protection scheme, 50 ms was the protection switching time. Since the fiber bus protection, the p-cycle scheme, the pre-configured *K*&*K* structure scheme, and the MPP scheme only need to conduct protection switching at the source and destination nodes, the recovery time was close to 50 ms. However, both the protection scheme and the restoration scheme were based on network connectivity that strictly required connectivity between any two nodes in a network. In other words, these two schemes worked as long as the network between the source–destination node pair of a connection was still connected after a natural or man-made disaster. If multi-failure or a natural disaster break the network into several disconnected parts, these technologies are useless. 

For each developed survivability technology, we had to first ensure the required survivability requirements were met, and then aim to minimize the total backup spectrum resource consumption. For the fiber bus protection scheme, self-healing architecture, and 1 + 1 protection scheme, the consumed backup spectrum resources were almost equal to the working spectrum resources. The photonic millimeter-wave bridge scheme mainly consumed the wireless resources. Table 2 presents the technologies that are used to minimize the backup spectrum allocation of existing survivability technologies including the p-cycle scheme, the pre-configured *K*&*K* structure scheme, the multi-path protection scheme, and the restoration scheme. From Table 2, we can see that the ILP model is a widely used method to minimize total backup spectrum resource consumption. However, the ILP model can only be applied to small-scale networks. Therefore, various heuristic algorithms such as genetic and greedy algorithms, were applied. 

When establishing backup light paths, the spectrum allocated along the backup paths must satisfy the spectrum continuity and spectrum contiguity constraints in elastic optical networks or wavelength continuity constraints in WDM networks. Table 3 presents the technologies that are used to realize routing and spectrum allocation of existing survivability technologies. From Table 3, we can see that the routing and spectrum allocation constraints in backup paths can be easily implemented using the ILP model. Since the ILP model can only be applied to small-scale networks, other heuristic algorithms such as Dijkstra’s algorithm, KSP algorithm, genetic algorithm, greedy algorithm, wavelength plane, and first-fit have been applied.

Each kind of survivability technology has its applicable topology. Table 4 presents the applicable topologies of each kind of survivability technology. Since the pre-configured *K*&*K* structure scheme and the multi-path protection scheme are developed to resist multiple failures, the type of applicable topology was mesh. The 1 + 1 protection scheme had a wide application scope including mesh, ring, star, and line. The p-cycle scheme was designed for mesh networks. 

## 3. Sensing Connectivity and K-Node (Edge) Sensing Connectivity

In this section, we first elaborate on the disaster-resilient survivability requirements of optical sensing and optical communication in OSNs. Then, the concepts of content connectivity and *k*-node (edge) content connectivity are introduced. The *k*-node (edge) content connectivity was used to quantitatively measure content connectivity and achieve disaster-resilience. Based on *k*-node (edge) content connectivity, a new concept of *k*-node (edge) sensing connectivity, which ensures the connectivity between sensing data and users, is proposed. Moreover, the key technologies of implementing *k*-node (edge) sensing connectivity are also elaborated. 

### 3.1. Disaster-Resilient Survivability Requirement

Due to wide distribution and high severity, natural disasters are big threats to normal operation of OSNs. Natural disasters typically and frequently occur in countries with large territories, such as Russia, Canada, China, and the USA. Using China as an example, the types of natural disasters mainly include earthquake, landslide, mud-rock flow, flood, volcano, rainstorm, land freezing and thawing, snowstorm, altitude cold, and high temperature. Nowadays, the quality of the global environment continues to deteriorate and natural disasters occur frequently. For example, in 2008, the 7.8-magnitude Wenchuan earthquake in China led to around 30,000 km of cut fiber optic cables and 4000 telecom offices becoming ineffective. Rescuers could only use satellite to contact the outside world, which brought great difficulty to rescue work. Consequently, the disaster-resilient sensing and survivability of OSNs has attracted more and more attention. Table 5 presents the characteristics of different kinds of natural disasters. A disaster having wide distribution, high probability, and high severity can seriously influence the normal operation of OSNs. Therefore, all disasters can be divided into several groups according to their degree of influence. To reduce the influence of natural disasters, we defined the following regulations of light-path protection and data center backup. These regulations can help design disaster-resilient OSNs. 

(1) For natural disasters with a wide affected region, low probability, and high severity, all working and backup light paths should avoid passing through a common disaster area.

(2) For natural disasters with a narrow affected region, high probability, and low severity, the number of passing disaster areas of a working light path or its backup light paths should not exceed a given upper bound.

(3) For natural disasters with a wide affected region, low probability, and low severity, the number of passing disaster areas of a working light path or backup light paths should not exceed a given upper bound.

(4) For natural disasters with a wide affected region, high probability, and low severity, all light paths and data centers should avoid passing through a common disaster zone. 

### 3.2. Content Connectivity and K-Node (Edge) Content Connectivity

**Figure 7:**
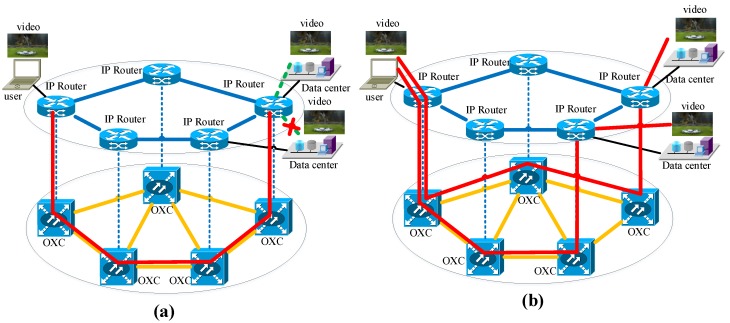
Network and content connectivity: (**a**) video service provisioning through one end-to-end light path; (**b**) video service provisioning through end-to-content light paths; OXC: optical cross connect equipment.

The content connectivity is proposed with respect to network connectivity. Network connectivity is one of the basic concepts of graph theory. It is an important measure of the robustness of a network. Network connectivity indicates the minimum number of elements (nodes or edges) that need to be removed to disconnect the remaining nodes from each other. Based on this, content connectivity is defined as the reachability of the content from any point of a data center network [36]. Figure 6 presents the survivability capability comparison between network connectivity and content connectivity. In Figure 7a, a user obtains the required video through one end-to-end light path. However, the video is interrupted when a failure occurs on any link along this light path. In Figure 7b, a user obtains the required video through end-to-content light paths. Even if a failure occurs on any link in physical layer, the video is still available. The content connectivity has been used to improve the survivability of optical data center networks against natural disasters. In [127], the authors proposed a protection scheme for a disaster recovery center based on content connectivity against disasters in disaster recovery center networks. In [128], the authors proposed survivable multipath routing and spectrum allocation with a content connectivity scheme in OFDM-based elastic data center networks. In [129], the authors focused on double-link failures and considered different combinations of content connectivity and network connectivity. They presented an ILP formulation for survivable virtual network mapping to guarantee the network connectivity after single-link failures to maintain the content connectivity after double-link failures. In [130], the authors proposed a dynamic content replacement scheme to minimize spectrum consumption, blocking probability, and network latency. In [131], the authors proposed an efficient dynamic content replacement scheme to minimize blocking probability and resource utilization. In [132], the authors proposed a disaster-aware dynamic content-management algorithm that can adjust the existing placement based on dynamic settings. Besides reducing the overall risk and making the network disaster aware, reducing network resource usage and satisfying quality-of-service requirements can also be achieved via this approach. In graph theory, *k*-node (edge) network connectivity is defined where there does not exist a set of *k* − 1 nodes (edges) whose removal disconnects the connectivity from any remaining node to the needed content, which is replicated and maintained in multiple data centers. In contrast to *k*-node (edge) network connectivity, *k*-node (edge) content connectivity is defined where there does not exist a set of *k* − 1 nodes (edges) whose removal disconnects the connectivity from any remaining node to the needed content, which is replicated and maintained in multiple data centers [37]. The *k*-node (edge) content connectivity can be used to design disaster-resilient optical data center networks. In [133,134], the authors proposed a shared protection scheme based on the sharing principle among multiple end-to-content backup light paths in optical data center networks. Numerical results showed that it can reduce wavelengths consumption while ensuring the survivability against multiple failures. In [135], the authors developed an ILP model and heuristic algorithms to design disaster-resilient *k*-node (edge) content connected optical data center network. In [136], the authors conducted distance-adaptive RSA for end-to-content light paths in *k*-node (edge) content connected elastic optical data center networks.

### 3.3. K-Node (Edge) Sensing Connectivity 

The definition of *k*-node (edge) content connectivity describes the relationship between content connectivity and the node (edge) set, which might be simultaneously affected by one disaster event. The *k*-node (edge) content connectivity of optical data center networks becomes an important measure of its survivability against natural disasters. Menger’s theorem is the foundation of network connectivity. In contrast to *k*-node (edge) content connectivity, the concept of *k*-node (edge) sensing connectivity, which ensures the connectivity between sensing data and users, is proposed for OSNs. The *k*-node (edge) sensing connectivity can be defined where there does not exist a set of *k* − 1 nodes (edges) whose removal disconnects the connectivity from any collector to the required sensing data, which is replicated and maintained in multiple sensors. An OSN is called *k*-node (edge) sensing connected when it satisfies *k*-node (edge) sensing connectivity requirements. We can achieve *k*-node (edge) sensing-connected optical sensor networks by searching independent *k* end-to-sensing light paths between a collector and multiple sensors where the sensing data is hosted [37]. Multiple end-to-sensing light paths are independent if they do not have any internal node in common. The *k*-node (edge) sensing connectivity has the advantage of sensing backup, multi-path routing, and disaster-resilience. 

**Sensing Backup**: Sensing data is replicated and maintained in multiple sensors. Multiple sensors, which are chosen as candidate sensors for sensing data deployment, can be required to be disaster-disjointed. 

**Multi-Path Routing**: There exist at least *k* independent end-to-sensing light paths that can be used by each collector to access the sensing data. Generally, the value of *k* is greater than the upper bound of network connectivity. 

**Disaster-Resilience**: The end-to-sensing light paths are independent and terminate at different sensors. The probability of sensing data inaccessibility caused by a disaster event can be significantly reduced. The end-to-sensing light paths can also be designed to be disaster-disjointed. 

The *k*-node (edge) sensing connectivity can be achieved by searching *k* independent light paths between users and multiple sensors where the sensing data is hosted. The realization of *k*-node (edge) sensing-connected OSNs can be decomposed into three sub-problems: flexible sensor deployment, maximum number of independent end-to-sensing light path calculations, and the routing and spectrum allocation (RSA) for *k* end-to-sensing light paths [135].

**Flexible Sensor Deployment**: The sensor set is chosen where the sensing data can be obtained. There are three strategies: spectrum-aware sensor deployment, capacity-aware sensor deployment, and disaster-aware sensor deployment. Each deployment strategy can be adopted for different network scenarios and requirements.

**Maximum Number of Independent End-to-Sensing Light Path Calculations**: The maximum number of independent end-to-sensing light paths is searched between users and multiple sensors where the sensing data are obtained. It can also be used to determine if current sensor deployment satisfies *k*-node (edge) sensing connectivity requirements.

**The RSA for *K* End-to-Sensing Light Paths**: In elastic optical networks, we need to address the problem of RSA for each end-to-sensing light path. The RSA for each end-to-sensing light path must satisfy spectrum contiguity and spectrum continuity requirements.

#### 3.3.1. Flexible Sensor Deployment

Sensor deployment must first ensure that each collector has *k* independent light paths to access sensing data. A lower number of repetitions per sensing data is better. We constructed a set of candidate sensors by greedily choosing the sensor with the minimum cost from available sensors. The cost of sensors can be evaluated via different strategies. When all users have at least *k* independent end-to-sensing light paths to candidate sensor sets, this candidate sensor set is the final result. The followings are three optional sensor deployment strategies: 

**Spectrum-Aware Sensor Deployment:** The sensor whose adjacent links have a large number of available spectrum resources is the preferred choice as a candidate sensor for sensor deployment.

**Capacity-Aware Sensor Deployment:** The deployment area with a large remaining capacity is the preferred choice as a candidate sensor for deployment. In this situation, the capacity of the deployment area plays a more important role than the spectrum resource. The network operator should adopt this strategy when the current optical sensor network has enough spectrum resources compared with capacity resources in the deployment area. 

**Disaster-Aware Sensor Deployment:** In order to avoid a disaster event destroying all sensors where the sensing data is obtained, the sensor must be placed in a more reliable area. The sensor that is far from the area where the natural disasters occur frequently is the preferred choice as a candidate sensor. While researchers in climatology, geology, and environmental science have been studying how to predict disasters and assess disaster risks for certain regions, networking research can exploit this information to develop novel methods to prepare networks to handle disasters with the knowledge of risky regions, to better prepare them for a predicted disaster. 

#### 3.3.2. Maximum Number of Independent End-to-Sensing Light Path Calculations

**Figure 8:**
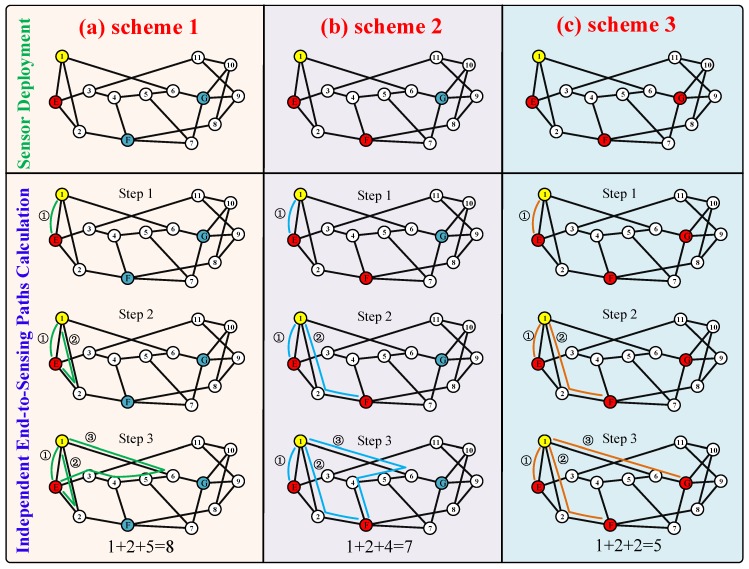
Independent end-to-sensing light path calculations: (**a**) independent end-to-sensing light paths with one available sensor; (**b**) independent end-to-sensing light paths with two available sensors; (**c**) independent end-to-sensing light paths with three available sensors.

*K*-node (edge) sensing connectivity means that at least *k* independent paths exist and can be used by each collector to access the sensing data. An OSN is called *k*-node (edge) sensing-connected when it satisfies *k*-node (edge) sensing connectivity requirements. Therefore, we can achieve *k*-node (edge) sensing-connected OSNs by searching for independent *k*. To determine if the current sensor deployment satisfies the *k*-node (edge) sensing connectivity requirement or not, we need to search the maximum number of independent end-to-sensing light paths between users and the sensors set from current sensor deployment. If the maximum number of independent end-to-sensing light paths between the collector and the sensors set from current sensor deployment is greater than or equal to *k*, we believe that the candidate sensor set satisfies the *k*-node (edge) sensing-connected requirement. The algorithm that searches the maximum number of independent end-to-sensing light paths between the node and node set can be proved to be Non-deterministic Polynomial Complete (NP-Complete). The ILP model and heuristic methods, such as ant colony, genetic algorithm, and simulated annealing algorithm, can be used to obtain the maximum number of independent end-to-sensing light paths. Figure 8 presents the schematic diagram of the maximum number of independent end-to-sensing light path calculations. In Figure 8a–c, the number of available sensors is one, two, and three, respectively. For example, in Figure 8c, the maximum number of independent end-to-sensing light paths between user_1 and sensors E, F, and G is three. These three light paths are P_1_: 1→E, P_2_: 1→2→F, and P_3_: 1→6→G. 

#### 3.3.3. The RSA for End-to-Sensing Light Paths

**Figure 9:**
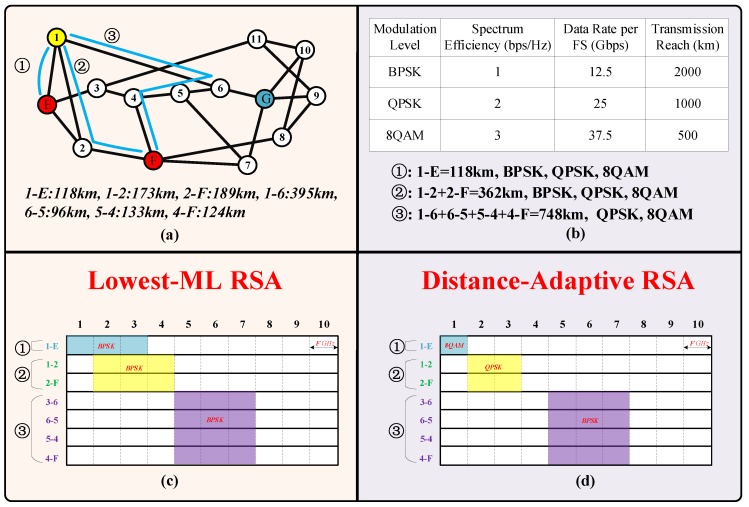
The RSA for end-to-sensing paths: (**a**) end-to-sensing paths; (**b**) the transmission distance and capacity of each kind of modulation level; (**c**) the lowest modulation level allocation; (**d**) the distance-adaptive modulation level allocation.

In elastic optical networks, we need to address the problem of RSA for each end-to-sensing light path. The RSA for each end-to-sensing light path must satisfy spectrum contiguity and spectrum continuity requirements. The spectrum contiguity constraint requires all the Frequency Slots (FSs) assigned to an end-to-sensing light path to be spectrally neighboring. The spectrum continuity constraint requires that all the fiber links traversed by an end-to-sensing light path use the same set of FSs. We assumed that the transponder at each requesting node was tunable, so any end-to-sensing light path can use different sets of contiguous FSs. The modulation levels can affect the QoT and the transmission distance of the light path. The spectrum can also affect the QoT and can influence the resource efficiency of OSNs. In Figure 9a, the content is replicated and maintained in sensor E and sensor F in NSFNet. Three independent end-to-sensing light paths are established to design a three-node (edge) sensing-connected sensor network. The three end-to-sensing light paths are P_1_: 1→E, P_2_: 1→2→F, and P_3_: 1→6→5→4→F. The transponder at requesting node_1 was tunable, so the light paths P_1_, P_2_, and P_3_ used different sets of contiguous FSs. As presented in Figure 9b, the light path P_1_ with length 118 km used the modulation level BPSK, QPSK, and 8QAM. Figure 9c,d compare the consumed spectrum between lowest Modulation Level-Routing Spectrum Allocation (ML-RSA) light path and distance-adaptive RSA. The lowest ML-RSA chose the lowest modulation level for any end-to-sensing light path. Distance-adaptive RSA chose the appropriate modulation level according to the length of the end-to-sensing light path. The comparison shows that the distance-adaptive RSA presented higher spectrum efficiency. 

## 4. AI-Assisted Sensing and Survivability Techniques

Recently, AI has become increasingly important and many countries have taken it as the future research focus for the next few years. AI has been applied in many aspects of optical networks. In [38], the authors gave an overview of the application of machine learning in optical communications and networking. The current research involves failure localization and anomaly detection, routing and resource allocation, QoT estimation, etc. Moreover, AI has been applied in vehicular networks, and vehicular edge and vehicular fog computing [137,138,139,140,141]. In all AI-assisted applications, the QoT estimation is an important one. In the field of optical sensing, AI also has important applications, including learning, reasoning, and self-correction.

### 4.1. AI-Assisted QoT Estimation

AI-Assisted QoT estimation not only guarantees the QoS for unicast and multicast services but also helps to improve the survivability of optical sensing and optical communication in optical data center networks. In [142], the authors used machine learning techniques to estimate the QoT of light paths in coherent uncompensated WDM links. In [143], the authors developed a machine learning classifier that predicted whether the bit error rate of unestablished light paths met the required system threshold based on traffic volume, desired route, and modulation format. In [144], the authors evaluated the effectiveness of various machine learning models used to predict the QoT of an unestablished light path. The considered models are: K-nearest neighbor, logistic regression, support vector machines, and artificial neural networks. In [145], the authors realized QoT prediction using machine learning for dynamic operation of optical WDM networks. The AI-assisted failure localization and anomaly detection can quickly and accurately identify the number of failures and the location of each failure. The QoT estimation can help to accurately determine if a failure occurs along a light path or not. These technologies provide prerequisites for the following protection and restoration schemes. In the future, AI can be used to search the optimal protection structure and recovery path, and conduct spectrum-efficient resource allocation. When a large number of working flows are interrupted by disasters, AI should help sort all interrupted working flows and give a reasonable recovery order. At the same time, the transmission capacity of optical fiber is improved gradually. Multiplexing technologies such as time division multiplexing TDM, WDM, and space division multiplexing (SDM) are applied to optical communication to extend transmission capacity [146,147]. AI can help improve the survivability of optical sensing in large-capacity OSNs. 

### 4.2. AI-Assisted Sensing on Learning

Since sensors have limited range and coverage, mobile robots often have to make decisions on where to point their sensors. A good sensing strategy allows a robot to collect information that is useful for its tasks. In [148], the author showed how to learn active sensing strategies for a mobile robot using least square policy iteration, and experimental results suggested their approach was able to learn highly effective sensing strategies. Spectrum sensing plays a key role for opportunistic spectrum access in cognitive radio networks (CRN). Two main sensing schemes consist of individual spectrum sensing, in which each secondary user (SU) makes sensing decisions independently, and cooperative spectrum sensing, in which multiple SUs exchange their sensing information with the fusion center, which makes the final decision. In [149], the author proposed novel cooperative spectrum sensing (CSS) algorithms for cognitive radio (CR) networks based on machine learning techniques, which were used for pattern classification, and concluded that the weighted KNN classifier was well suited for CSS, which required the updating of training energy vectors on-the-fly. In [150], the author proposed a low-dimensional probability vector as the feature vector for machine-learning-based classification, instead of the N-dimensional energy vector, in a CRN with a single primary user (PU) and N secondary users (SUs); due to its lower dimension, the probability vector based classification was capable of having a smaller training duration and a shorter classification time for testing vectors. With the fast advances in mobile devices and communication technologies, various machines and devices are capable of interacting with each other within a network, that is, the IoT. In [151], the author presented a general deep learning framework for RF sensing in the IoT, along with several experimental case studies, and a discussion of challenges and open problems. Sensor-equipped smartphones and wearables are transforming a variety of mobile apps ranging from health monitoring to digital assistants. In [152], the author investigated the potential for techniques from deep learning to address a number of critical barriers to mobile sensing surrounding inference accuracy, robustness, and resource efficiency.

### 4.3. AI-Assisted Sensing on Reasoning

Representation and reasoning concerning actions is a basic component for the design of cognitive robots. In [153], the author developed a formalism that allowed for sensing in reasoning about actions under qualitative and probabilistic uncertainty, thus formulating and addressing the problem of conditional planning under qualitative and probabilistic uncertainty. The distributed autonomous robotic system (DARS) can adapt to the variations of the environment and enhance the efficiency of system operation by the cooperation of multiple robots. In [154], the author addressed a problem of distributed sensing in the distributed autonomous robotic system (OARS) and concluded that evidential reasoning based on the Dempster–Shafer theory was suitable for the processing of distributed sensing. There has been an attempt to reconcile the theoretical work on reasoning about action with the realization of agents, in particular mobile robots. In [155], a logical framework for representing dynamic systems based on description logics is presented, which allowed for the formalization of sensing actions. Adaptive systems are expected to adapt to unanticipated run-time events using imperfect information about themselves, their environment, and goals. In [156], the paper contributed a formal analysis technique that explicitly considered uncertainty in sensing when reasoning the best way to adapt, together with uncertainty reduction mechanisms to improve system utility. Sensorless robotic assembly requires the use of hard automation to eliminate the effect of real life uncertainty. In [157], a method for determining sensing requirements for robotic assemblies from a geometrical analysis of critical contact-state transitions produced among mating parts during the execution of nominal assembly plans was presented.

### 4.4. AI-Assisted Sensing on Self-Correction

In the big data era, it is important to identify trustworthy information from an influx of noisy data contributed by unvetted sources from online social media (e.g., Twitter, Instagram). This task is referred to as truth discovery, which aims at identifying the reliability of the sources and the truthfulness of claims they make without knowing either of them a priori. In [158], the paper presented a new robust approach to solve the truth discovery problem in social media sensing applications, and the approach had a self-correction function. The barriers for adopting robotic technology in the fettling process arise from the cost factor and technical difficulties in programming robots to handle the widely varying nature of parting lines and the non-uniform distribution of unwanted material around the molded casting. In [159], the authors presented the results and implications of their experimental study into robotic fettling operations using a visual feedback technique and force sensing, and this approach gave better accuracy and efficiency in terms of self-correction.

### 4.5. Statistical Analysis

AI is a collective name, covering a variety of individual technologies such as decision tree, association rules, support vector machine (SVM), artificial neural networks (ANNs), Bayesian networks, genetic algorithms, and k-nearest neighbor (KNN). Among these technologies, ANNs inspired by biological neural networks have a powerful nonlinear modeling ability when introducing hidden layers. Table 6 presents the applied AI techniques in optical sensing on learning, reasoning, and self-correction. It can be seen that ANN is the most widely used AI technique. 

## 5. Conclusions

This paper reviews and evaluates the traditional survivability technologies for optical sensing and optical communication in OSNs. The existing survivability technologies can be divided into two categories: protection and restoration. The protection schemes such as the fiber bus protection scheme, the self-healing scheme, 1 + 1 protection scheme, the p-cycle scheme, the p-polyhedron scheme, and the multipath protection scheme have low time consumption but high spectrum resource consumption. Compared with the protection scheme, the restoration scheme has longer recovery time and cannot guarantee 100% recovery. Moreover, since the RSA problem has been proved to be an NP-complete problem, various heuristic algorithms and biological intelligence algorithms have been applied to spectrum allocation for backup light paths. Since the traditional survivability technologies are based on network connectivity, it is difficult to achieve disaster-resilience by merely relying on an optical network itself. The photonic millimeter-wave bridge scheme uses millimeter-wave technology to recover the interrupted traffic. This scheme does not rely on an optical network itself and thus provides a promising method to achieve disaster-resilience. In addition, the concept of *k*-node (edge) sensing connectivity is introduced. Based on *k*-node (edge) sensing connectivity, disaster-resilient survivability technologies are elaborated for OSNs. Recently, AI has obtained rapid development. It provides a pre-requisite for traditional survivability technologies and can help improve the survivability of optical sensing and optical communication in OSNs. AI has been applied in many fields of optical communication and optical sensing including learning, reasoning, and self-correction. In the future, other fields such as sensor networking, management and operation of OSN, can also be addressed by AI.

## Figures and Tables

**Table 1:** Performance comparisons among traditional survivability technologies.

Survivability Technology	Survival Capability	Recovery Time	Resource Efficiency	Complexity
The Fiber Bus Protection Scheme [40,41,42,43,44,45]	Single-Failure	50 ms	low	low
The Self-Healing Architecture [46,47,48,49,50,51,52,53]	Single-Failure	>50 ms	low	low
The 1 + 1 Protection Scheme and Extensions [56,57,58,59,60,61,62,63,64,65,66,67,68,69,70,71,72]	Single-Failure	~50 ms	low	low
The Photonic Millimeter-Wave Bridge Scheme [73,74,75,76,77,78]	Single-Failure	>50 ms	low	high
The P-Cycle Scheme [79,80,81,82,83,84,85,86,87,88,89,90,91,92,93,94,95,96,97]	Single-Failure	~50 ms	high	high
The Pre-Configured *K*&*K* Structure Scheme [98,99,100,101,102,103,104,105,106,107,108,109,110]	Multi-Failures	~50 ms	high	high
The Multi-Path Protection Scheme [111,112,113,114,115,116,117,118,119,120]	Multi-Failures	~50 ms	low	high
The Restoration Scheme [121,122,123,124,125,126]	Single-Failure/Multi-Failures	>50 ms	high	high

**Table 2:** Technologies used to minimize backup spectrum allocation.

Survivability Technology	Technologies
The 1 + 1 Protection Scheme and Extensions	Two-Step Searching [56], Signal Overlap [58], Network Coding [59], The ILP Formulation [61,67,68,69,70], Higher-Order Modulation Format [67,70,72], Minimum Free Spectrum–Block Consumption [68],
The P-Cycle Scheme	The ILP Formulation [79,80,81,83,85,86,88,89,93,94,95,96,97] Genetic Algorithm [81,91], Efficiency Score [82,83,92,93], The ILP-Genetic Algorithm [90]
The Pre-Configured *K*&*K* Structure Scheme	The ILP Formulation [98,99,101,104,105,106,107,109,110], Greedy Algorithm [105]
The Multi-Path Protection Scheme	Reconfiguration [111,112], The ILP Formulation [112,113,114,115,116,117,118,119,120]
The Restoration Scheme	The ILP Formulation [124]

**Table 3:** Technologies used to conduct routing and spectrum allocation in backup paths.

Survivability Technology	Routing	Spectrum Allocation
The 1 + 1 Protection Scheme and Extensions	Dijkstra’s Algorithm [56,67,70,71,72], The ILP Formulation [58,59,61,67,68,69,70,72], KSP Algorithm [68,69]	Wavelength Plane [56], The ILP Formulation [58,59,61,67,68,69,70,72], Spectrum Window Plane [61,67,70,71,72], Available Spectrum Block [68], First-Fit [69]
The P-Cycle Scheme	The ILP Formulation [79,80,81,83,85,86,88,89,93,94,95,96,97], Straddling Link Algorithm [79,93], Genetic Algorithm [81,91], Distinct K-Shortest Path Algorithm [82], Protection Cardinality of Demand [83], The ILP-Genetic Algorithm [90], Breadth-First Search [92]	The ILP Formulation [79,80,81,83,85,86,88,89,93,94,95,96,97], Genetic Algorithm [81,91], The ILP-Genetic Algorithm [90]
The Pre-Configured *K*&*K* Structure Scheme	The ILP Formulation [98,101,104,105,106,107,109,110], Cycle Extension [98,99,103], Depth-First Search Algorithm [102], Greedy Algorithm [105], Disjoint Paths Algorithm [108]	The ILP Formulation [98,99,101,104,105,106,107,109,110], Maximum Spare Capacity [103],
The Multi-Path Protection Scheme	Bhandari’s link-disjoint Paths Algorithm [111,112,113,117], The ILP Formulation [112,113,114,115,117,118,119,120], KSP Algorithm [115,119],	First-Fit [111,112,113,115,119], The ILP Formulation [112,113,114,115,117,118,119,120], Genetic Algorithm [117]
The Restoration Scheme	KSP Algorithm [121], The ILP Formulation [124], KSP [124]	First-Fit [121], The ILP Formulation [124]

**Table 4:** The applicable topologies of each kind of survivability technology.

Survivability Technology	Applicable Topologies
The Fiber Bus Protection Scheme	Line [40,41,42,43,44,45]
The Self-Healing Architecture	Ring [46,47,48,50], Ring-Mesh [49] Two-Level Ring [51], Star-Ring-Bus [52], Star-Ring [53]
The 1 + 1 Protection Scheme and Extensions	Mesh [56,58,59,61,62], Ring [57,66,67,68,69,70,71,72], Star [60,63], Line [64,65]
The Photonic Millimeter-Wave Bridge Scheme	Line [75,78], Star [73,74,76,77]
The P-Cycle Scheme	Mesh [79,80,81,82,83,84,85,86,87,88,89,90,91,92,93,94,95,96,97]
The Pre-Configured *K*&*K* Structure Scheme	Mesh [98,99,100,101,102,103,104,105,106,107,108,109,110]
The Multi-Path Protection Scheme	Mesh [111,112,113,114,115,116,117,118,119,120]
The Restoration Scheme	Mesh [121,122,123,124,125]

**Table 5:** The characteristics of different kinds of natural disasters.

Disasters	Affect Region	Probability	Severity	Impact
Earthquake	Wide	Low	High	Fiber Line/Sensors
Landslide	Narrow	High	Low	Fiber Line/Sensors
Collapse	Narrow	High	Low	Fiber Line
Mud–Rock Flow	Narrow	High	Low	Fiber Line
Flood	Wide	Low	Low	Fiber Line/Sensors
Volcano	Narrow	Low	Low	Fiber Line/Sensors
Rainstorm	Wide	High	Low	Fiber Line/Sensors
Land Freezing and Thawing	Narrow	High	Low	Fiber Line
Snowstorm	Wide	Low	Low	Fiber Line
High Temperature	Wide	High	Low	Sensors

**Table 6:** The applied AI techniques in optical sensing on learning, reasoning, and self-correction.

AI-Assisted Sensing Techniques	Applied AI Techniques
AI-Assisted Sensing on Learning	Ordinary Least Squares Regression [148], KNN [149], SVM [150], RF [151], ANN [152]
AI-Assisted Sensing on Reasoning	Bayesian [153,157], ANN [154], Logistic Regression [155], Decision tree [156]
AI-Assisted Sensing on Self-Correction	SVM [158], Decision tree [159]
